# *MAPT* subhaplotypes in corticobasal degeneration: assessing associations with disease risk, severity of tau pathology, and clinical features

**DOI:** 10.1186/s40478-020-01097-z

**Published:** 2020-12-07

**Authors:** Rebecca R. Valentino, Shunsuke Koga, Ronald L. Walton, Alexandra I. Soto-Beasley, Naomi Kouri, Michael A. DeTure, Melissa E. Murray, Patrick W. Johnson, Ronald C. Petersen, Bradley F. Boeve, Ryan J. Uitti, Zbigniew K. Wszolek, Dennis W. Dickson, Owen A. Ross, Michael G. Heckman

**Affiliations:** 1 Department of Neuroscience, Mayo Clinic, 4500 San Pablo Road, Jacksonville, FL 32224 USA; 2 Division of Biomedical Statistics and Informatics, Mayo Clinic, 4500 San Pablo Road, Jacksonville, FL 32224 USA; 3 Department of Neurology, Mayo Clinic, Rochester, MN USA; 4 Department of Neurology, Mayo Clinic, Jacksonville, FL USA; 5 Department of Clinical Genomics, Mayo Clinic, Jacksonville, FL USA

**Keywords:** Corticobasal degeneration, *MAPT*, Genetics, Neuropathology

## Abstract

The microtubule-associated protein tau (*MAPT*) H1 haplotype is the strongest genetic risk factor for corticobasal degeneration (CBD). However, the specific H1 subhaplotype association is not well defined, and it is not clear whether any *MAPT* haplotypes influence severity of tau pathology or clinical presentation in CBD. Therefore, in the current study we examined 230 neuropathologically confirmed CBD cases and 1312 controls in order to assess associations of *MAPT* haplotypes with risk of CBD, severity of tau pathology (measured as semi-quantitative scores for coiled bodies, neurofibrillary tangles, astrocytic plaques, and neuropil threads), age of CBD onset, and disease duration. After correcting for multiple testing (*P* < 0.0026 considered as significant), we confirmed the strong association between the *MAPT* H2 haplotype and decreased risk of CBD (Odds ratio = 0.26, *P* = 2 × 10^−12^), and also observed a novel association between the H1d subhaplotype and an increased CBD risk (Odds ratio = 1.76, *P* = 0.002). Additionally, although not statistically significant after correcting for multiple testing, the H1c haplotype was associated with a higher risk of CBD (Odds ratio = 1.49, *P* = 0.009). No *MAPT* haplotypes were significantly associated with any tau pathology measures, age of CBD onset, or disease duration. Though replication will be important and there is potential that population stratification could have influenced our findings, these results suggest that several *MAPT* H1 subhaplotypes are primarily responsible for the strong association between *MAPT* H1 and risk of CBD, but that H1 subhaplotypes are unlikely to play a major role in driving tau pathology or clinical features. Our findings also indicate that similarities in *MAPT* haplotype risk-factor profile exist between CBD and the related tauopathy progressive supranuclear palsy, with H2, H1d, and H1c displaying associations with both diseases.

## Introduction

Corticobasal degeneration (CBD) is a rare and progressive neurodegenerative disorder, with an estimated prevalence of approximately 6 cases per 100,000 people [12]. Age of disease onset is most commonly between 40 and 70 years, and the average survival time is 7 years [12]. Patients with CBD can present with a variety of clinical features which overlap with other neurodegenerative disorders, including progressive supranuclear palsy (PSP), Alzheimer’s disease (AD), and frontotemporal dementia (FTD), which makes clinical diagnosis challenging [2, 5, 12]. Indeed, a definitive diagnosis of CBD can only be made neuropathologically, where CBD is characterized by deposition of tau in neurons and glia in the form of pretangles, neurofibrillary tangles (NFT), neuropil threads (NT), astrocytic plaques (AP), and oligodendroglial coiled bodies (CB) [7]. Tau in CBD is composed disproportionately of isoforms with four repeats in the microtubule binding domain (4R tau) in the tau gene (*MAPT*) [4]. While PSP and CBD are both primary 4R-tauopathies, tau pathology occurs disproportionately in forebrain structures in CBD and in hindbrain structures in PSP, although both regions are affected in both disorders [12, 18].

Due to its rarity, little is known about the genetics of CBD [20]. Initial studies of common genetic variation in CBD implicated the *MAPT* H1 haplotype [6, 10, 17, 25]. These findings were subsequently confirmed in the first genome-wide association study (GWAS) of CBD [11], where the H1 haplotype was identified as the strongest genetic risk factor (Odds ratio [OR] = 3.70, *P* = 1.4 × 10^−12^), with the rs242557 variant which partially tags the H1c subhaplotype also displaying some evidence of association with CBD risk (OR = 1.57, *P* = 7.9 × 10^−6^). The *MAPT* gene is characterized by two main haplotypes related to a large inversion on chromosome 17, whereby the H1 haplotype is more common and the H2 haplotype is rarer and virtually absent for individuals of non-European backgrounds [21]. The H1 haplotype can be further categorized into more than 20 common subhaplotypes, including H1c and others [17]. Notably, a recent study observed associations of several *MAPT* subhaplotypes with risk of PSP, and to a lesser extent with severity of tau pathology in PSP [9]. The clinical and neuropathological similarities between PSP and CBD, as well as evidence that indicates that these two tauopathies have significant shared genetic risk [26], suggests that H1 subhaplotypes could also play a role in CBD. However, there has been no study to date of notable sample size that has examined how *MAPT* subhaplotypes may influence susceptibility to CBD or clinical and neuropathological features of CBD. Therefore, the aim of the current study was to examine associations of *MAPT* subhaplotypes with risk of CBD, tau pathology severity, and clinical features in this rare and understudied neurodegenerative disorder.

## Materials and methods

### Study patients

This study included a total of 230 neuropathologically-confirmed CBD cases and 1312 clinical controls. Of note, 139 of the CBD cases were included in the aforementioned CBD GWAS (72 in the discovery stage, and 67 in the replication stage) [11]. The CBD cases were obtained from the Mayo Clinic brain bank for neurodegenerative disorders between 1998 and 2019. All cases were examined by a single neuropathologist (DWD) using standardized histopathologic methods and phospho-tau immunochemistry. Diagnosis of CBD was made using current neuropathologic criteria [7]. Controls were recruited from the Mayo Clinic in Jacksonville, Florida (N = 881) or Rochester, Minnesota (N = 431) and were free from neurological disease. Information regarding age of CBD onset and disease duration was available from medical records for 188 of the 230 CBD cases. Controls were in part collected through the Mayo Clinic Study of Aging (MCSA), Alzheimer’s disease Research Center (ADRC), and the Mayo Clinic Udall Parkinson’s Disease Research Center of Excellence. All subjects were unrelated non-Hispanic Caucasians. Characteristics of CBD cases and controls are detailed in Table 1.

**Table 1:** Characteristics of CBD patients and controls

Variable	CBD patients (N = 230)	Controls (N = 1312)
Age (years)^1^	70 (46, 96)	69 (45, 92)
Sex		
Female	119 (51.7%)	611 (46.6%)
Male	111 (48.3%)	701 (53.4%)
Age of CBD onset (years)	64 (41, 86)	N/A
Disease duration (years)	6 (2, 16)	N/A
Braak stage		
0	27 (13.8%)	N/A
I	36 (18.4%)	N/A
II	66 (33.7%)	N/A
III	53 (27.0%)	N/A
IV	11 (5.6%)	N/A
V	2 (1.0%)	N/A
VI	1 (0.5%)	N/A
Thal phase		
0	105 (53.6%)	N/A
1	35 (17.9%)	N/A
2	24 (12.2%)	N/A
3	25 (12.8%)	N/A
4	3 (1.5%)	N/A
5	4 (2.0%)	N/A
CB overall tau pathology score	0.77 (0.23, 1.75)	N/A
NFT overall tau pathology score	2.19 (1.13, 2.62)	N/A
AP overall tau pathology score	0.53 (0.24, 1.04)	N/A
NT overall tau pathology score	2.52 (1.24, 2.95)	N/A

*CBD* corticobasal degeneration, *CB* coiled bodies, *NFT* neurofibrillary tangles, *AP* astrocytic plaques, *NT* neuropil threads

The sample median (minimum, maximum) is given for age. ^1^ Age represents age at death in CBD patients and age at blood draw in controls. Information was unavailable in CBD cases for age of CBD onset (N = 42), disease duration (N = 42), Braak stage (N = 34), Thal phase (N = 34), CB overall tau pathology score (N = 34), NFT overall tau pathology score (N = 34), AP overall tau pathology score (N = 34), and NT overall tau pathology score (N = 34)

### Genetic analysis

Total genomic DNA was extracted from peripheral blood lymphocytes in controls and from brain tissue in CBD cases using standard protocols [15]. A total of six *MAPT* variants (rs1467967, rs242557 [the H1c haplotype partial tagging variant], rs3785883, rs2471738, rs8070723 [the H2 haplotype tagging variant], and rs7521) were genotyped to examine *MAPT* haplotypes as previously described [17]. *MAPT* variants were genotyped using TaqMan SNP genotyping assays on an ABI 7900HT Fast Real-Time PCR system (Applied Bio systems, Foster City, CA, USA) according to manufacturer instructions (primer sequences available upon request). Genotypes were called using TaqMan Genotyper Software v1.3 (Applied Bio systems, Foster City, CA, USA). There were no deviations from Hardy–Weinberg equilibrium in controls (all *P* > 0.01 after Bonferroni correction), and genotype call rates were 100% for each variant. Genotype frequencies for each variant are summarized in Additional file 1: Table S1.

### Neuropathological assessment

For a subset of 196 CBD cases, a single neuropathologist (DWD) assessed semi-quantitative tau pathology measures on a 0–3 severity scale (0 = none; 1 = mild; 2 = moderate; 3 = severe). All sections were processed identically with phospho-tau monoclonal antibody (CP13, from Dr. Peter Davies, Feinstein Institute, Long Island, NY). Immunochemistry was performed using a DAKO Autostainer. CB, NFT, AP, and NT were evaluated and scored in 21 different neuroanatomical regions which are vulnerable to CBD pathology (Additional file 1: Table S2) [8]. For each of the four different tau pathology measures (CB, NFT, AP, and NT), one overall score was created by calculating the mean of the semi-quantitative scores (0, 1, 2, or 3) for each CBD case across all neuroanatomical regions, with a higher overall score indicating a greater severity of tau pathology (Table 1). In calculation of the four overall tau pathology scores, for CBD cases who did not have information available in a given region for a given tau pathology measure, these values were imputed by using the mean of the values of the CBD cases who did have this information available; this imputation was performed in order to avoid biasing overall tau pathology scores for CBD cases who did not have a tau pathology score in a neuroanatomical region (or regions) with generally less severe, or more severe, tau pathology. Any CBD cases who had missing data for greater than 50% of neuroanatomical regions for a given tau pathology measure were not included in analysis involving the overall score for that measure.

CBD cases were additionally assessed for Alzheimer type pathology with thioflavin S fluorescent microscopy. Braak NFT stage [3] and Thal amyloid phase [22] were assessed for each CBD case based on the density and distribution of plaques and tangles as previously described [16].

### Statistical analysis

Associations between six-variant *MAPT* haplotypes and risk of CBD were examined using score tests for association under a logistic regression framework [19], where tests were adjusted for age and sex. ORs and 95% CIs were estimated and are interpreted as the multiplicative increase in the odds of CBD corresponding to each additional copy of the given haplotype. In analysis of CBD cases, associations of six-variant *MAPT* haplotypes with overall CB, NFT, AP, and NT tau pathology scores, age of CBD onset, and disease duration were assessed using score tests for association under a linear regression framework [19]; tests were adjusted for age at death, sex, Braak stage, and Thal phase (analysis of tau pathology scores), for sex (analysis of age of CBD onset), and for sex and age of CBD onset (analysis of disease duration). Regression coefficients (β) and 95% CIs were estimated and are interpreted as the additive change in the mean outcome value corresponding to each additional copy of the given haplotype. Haplotypes that occurred in  < 1% of subjects in a given analysis were excluded for that analysis.

We utilized a Bonferroni correction for multiple testing, after which *P* values < 0.0026 (19 tests) were considered as significant when examining associations with risk of CBD and with overall tau pathology scores, and *P* values < 0.0028 (18 tests) were considered as statistically significant when assessing associations with age of CBD onset and disease duration. All statistical tests were two-sided. Statistical analyses were performed using R Statistical Software (version 3.6.2; R Foundation for Statistical Computing, Vienna, Austria).

## Results

Associations between six-variant *MAPT* haplotypes and risk of CBD are displayed in Table 2. After correcting for the 19 haplotypes that were examined (*P* < 0.0026 considered as significant), the H2 haplotype was strongly associated with a decreased risk of CBD (OR = 0.26, 95% CI: 0.18–0.38, *P* = 2 × 10^−12^), while the H1d subhaplotype was associated with increased CBD risk (OR = 1.76, 95% CI: 1.22–2.52, *P* = 0.002). Additionally, although not quite statistically significant, the H1c subhaplotype was also associated with a higher risk of CBD (OR = 1.49, 95% CI: 1.11–2.00, *P* = 0.009). Other nominally significant (*P* < 0.05) findings occurred for the H1b (OR = 1.31, 95% CI: 1.00–1.72, *P* = 0.049), and H1i (OR = 1.65, 95% CI: 1.01–2.70, *P* = 0.047) subhaplotypes.

**Table 2:** Associations between *MAPT* haplotypes and risk of CBD

Haplotype	*MAPT* variant						Haplotype frequency (%)		Association with CBD	
	rs1467967	rs242557	rs3785883	rs2471738	rs8070723	rs7521	CBD patients (N = 230)	Controls (N = 1312)	OR (95% CI)	*P* value
H1b	G	G	G	C	A	A	20.2	16.0	1.31 (1.00, 1.72)	0.049
H1c	A	A	G	T	A	G	15.2	11.3	1.49 (1.11, 2.00)	0.009
H1d	A	A	G	C	A	A	10.9	7.1	1.76 (1.22, 2.52)	0.002
H1e	A	G	G	C	A	A	7.5	9.0	0.92 (0.62, 1.36)	0.66
H1f	G	G	A	C	A	A	0.0	1.2	N/A^1^	0.14
H1g	G	A	A	C	A	A	1.6	1.1	1.45 (0.53, 3.96)	0.47
H1h	A	G	A	C	A	A	5.4	4.1	1.22 (0.72, 2.08)	0.47
H1i	G	A	G	C	A	A	5.9	4.4	1.65 (1.01, 2.70)	0.047
H1l	A	G	A	C	A	G	3.7	3.0	1.10 (0.61, 1.99)	0.75
H1m	G	A	G	C	A	G	1.6	2.9	0.77 (0.36, 1.64)	0.50
H1o	A	A	A	C	A	A	3.5	2.3	1.81 (0.93, 3.50)	0.080
H1p	G	G	G	T	A	G	0.9	1.5	0.82 (0.28, 2.43)	0.72
H1q	A	A	G	T	A	A	1.3	1.0	1.49 (0.57, 3.89)	0.42
H1r	A	G	G	T	A	G	2.6	1.1	2.03 (0.83, 4.93)	0.12
H1u	A	A	G	C	A	G	4.4	2.4	1.60 (0.87, 2.95)	0.13
H1v	G	G	A	T	A	G	1.7	1.2	1.10 (0.40, 3.03)	0.85
H1x	G	A	A	T	A	G	2.2	1.3	1.54 (0.64, 3.68)	0.34
H1y	A	A	A	T	A	G	0.5	1.6	0.54 (0.15, 1.95)	0.35
H2	A	G	G	C	G	G	7.1	22.7	0.26 (0.18, 0.38)	2 × 10^−12^

ORs, 95% CIs, and *P* values result from score tests of association that were adjusted for age and sex. ORs correspond to each additional copy of the given haplotype. *P* values < 0.0026 are considered as statistically significant after applying a Bonferroni correction for multiple testing

*CBD* corticobasal degeneration, *OR* odds ratio, *CI* confidence interval

^1^The H1f haplotype was not observed in CBD cases, and therefore estimation of an OR was not possible

When evaluating associations of *MAPT* haplotypes with CB, NFT, AP, and NT overall tau pathology scores in CBD cases (Table 3), no associations survived correction for multiple testing. Several nominally significant findings were observed (NFT: H1p [*P* = 0.021] and H1z [*P* = 0.033]; AP: H1e [*P* = 0.007]; NT: H1p [*P* = 0.028]), however none of these involved the H2, H1d, or H1c haplotypes that were associated with CBD risk. Similarly, there were no statistically significant (*P* < 0.0028 considered significant) associations between *MAPT* haplotypes and either age of CBD onset or disease duration (Table 4); the only nominally significant finding occurred for the rare H1p subhaplotype, which was associated with a longer disease duration (β = 3.42, 95% CI: 0.76–6.08, *P* = 0.013).

**Table 3:** Associations of *MAPT* haplotypes with overall tau pathology scores for coiled bodies, neurofibrillary tangles, tufted astrocytes, and neuropil threads

Haplotype	Haplotype frequency (%) (N = 196)	Association with CB overall tau pathology score		Association with NFT overall tau pathology score		Association with AP overall tau pathology score		Association with NT overall tau pathology score	
		β (95% CI)	*P* value	β (95% CI)	*P* value	β (95% CI)	*P* value	β (95% CI)	*P* value
H1b	20.7	− 0.05 (− 0.13, 0.02)	0.16	0.01 (− 0.06, 0.08)	0.79	− 0.03 (− 0.06, 0.00)	0.060	− 0.04 (− 0.11, 0.04)	0.31
H1c	14.6	0.08 (− 0.01, 0.16)	0.073	0.00 (− 0.07, 0.08)	0.96	0.02 (− 0.02, 0.06)	0.31	0.05 (− 0.04, 0.13)	0.26
H1d	10.8	0.02 (− 0.08, 0.11)	0.72	0.05 (− 0.04, 0.14)	0.28	0.01 (− 0.04, 0.05)	0.77	0.06 (− 0.04, 0.16)	0.23
H1e	7.4	0.01 (− 0.09, 0.12)	0.79	0.02 (− 0.08, 0.12)	0.71	− 0.07 (− 0.12, − 0.02)	0.007	− 0.07 (− 0.18, 0.04)	0.20
H1h	5.0	0.05 (− 0.08, 0.18)	0.48	− 0.06 (− 0.19, 0.06)	0.30	0.04 (− 0.02, 0.10)	0.23	0.01 (− 0.13, 0.14)	0.90
H1i	6.2	0.02 (− 0.11, 0.15)	0.82	0.09 (− 0.03, 0.21)	0.14	0.04 (− 0.02, 0.10)	0.22	0.09 (− 0.04, 0.22)	0.17
H1k^1^	1.0	0.33 (− 0.03, 0.68)	0.072	0.05 (− 0.28, 0.38)	0.78	0.02 (− 0.14, 0.18)	0.83	0.15 (− 0.28, 0.58)	0.51
H1l	3.4	0.00 (− 0.16, 0.16)	0.96	0.03 (− 0.12, 0.17)	0.73	− 0.03 (− 0.10, 0.05)	0.49	0.11 (− 0.05, 0.27)	0.18
H1m	1.5	− 0.09 (− 0.38, 0.20)	0.53	− 0.20 (− 0.47, 0.06)	0.13	0.11 (− 0.02, 0.24)	0.11	− 0.22 (− 0.51, 0.07)	0.14
H1o	4.6	− 0.09 (− 0.24, 0.07)	0.27	− 0.02 (− 0.17, 0.12)	0.74	0.03 (− 0.05, 0.10)	0.48	− 0.02 (− 0.17, 0.14)	0.84
H1p	1.0	− 0.12 (− 0.44, 0.21)	0.47	− 0.35 (− 0.64, − 0.05)	0.021	− 0.01 (− 0.15, 0.14)	0.95	− 0.37 (− 0.69, − 0.04)	0.028
H1q	1.2	− 0.10 (− 0.39, 0.18)	0.47	− 0.08 (− 0.34, 0.18)	0.54	0.07 (− 0.06, 0.20)	0.27	− 0.14 (− 0.42, 0.15)	0.34
H1r	2.3	− 0.09 (− 0.30, 0.13)	0.42	− 0.16 (− 0.35, 0.03)	0.11	− 0.04 (− 0.14, 0.05)	0.38	0.01 (− 0.20, 0.23)	0.90
H1s^2^	1.6	− 0.08 (− 0.34, 0.17)	0.51	0.08 (− 0.15, 0.32)	0.47	− 0.10 (− 0.21, 0.01)	0.089	0.14 (− 0.12, 0.39)	0.29
H1u	4.7	− 0.08 (− 0.21, 0.05)	0.25	0.10 (− 0.02, 0.22)	0.10	0.01 (− 0.05, 0.07)	0.65	0.06 (− 0.07, 0.19)	0.38
H1v	1.4	− 0.03 (− 0.29, 0.23)	0.83	− 0.05 (− 0.28, 0.19)	0.70	− 0.09 (− 0.21, 0.02)	0.12	− 0.20 (− 0.46, 0.06)	0.13
H1x	1.8	0.02 (− 0.21, 0.24)	0.87	0.10 (− 0.11, 0.31)	0.34	0.03 (− 0.08, 0.13)	0.61	0.04 (− 0.19, 0.27)	0.71
H1z^3^	1.0	0.02 (− 0.34, 0.39)	0.91	− 0.36 (− 0.69, − 0.03)	0.033	− 0.01 (− 0.17, 0.16)	0.95	− 0.28 (− 0.65, 0.09)	0.14
H2	7.9	0.02 (− 0.08, 0.12)	0.69	− 0.04 (− 0.13, 0.05)	0.42	0.03 (− 0.02, 0.07)	0.27	− 0.03 (− 0.13, 0.07)	0.59

β values, 95% CIs, and *P* values result from score tests of association that were adjusted for age at death, sex, Braak stage, and Thal phase. β values correspond to each additional copy of the given haplotype. *P* values < 0.0026 are considered as statistically significant after applying a Bonferroni correction for multiple testing

*CB* coiled bodies, *NFT* neurofibrillary tangles, *AP* astrocytic plaques, *NT* neuropil threads, *CBD* corticobasal degeneration, *β* regression coefficient, *CI* confidence interval

^1^The specific haplotype for H1k was not previously shown in Table 2 as this haplotype occurred at a frequency < 1% in the overall case–control series; this haplotype is A-A-A-C-A-G for the rs1467967-rs242557-rs3785883-rs2471738-rs8070723-rs7521 haplotype

^2^The specific haplotype for H1s was not previously shown in Table 2 as this haplotype occurred at a frequency < 1% in the overall case–control series; this haplotype is G-G-G-C-A-G for the rs1467967-rs242557-rs3785883-rs2471738-rs8070723-rs7521 haplotype

^3^The specific haplotype for H1z was not previously shown in Table 2 as this haplotype occurred at a frequency < 1% in the overall case–control series; this haplotype is G-A-G-T-A-G for the rs1467967-rs242557-rs3785883-rs2471738-rs8070723-rs7521 haplotype

**Table 4:** Associations of *MAPT* haplotypes with age of CBD onset and disease duration

Haplotype	Haplotype frequency (%) (N = 188)	Association with age of CBD onset		Association with disease duration	
		β (95% CI)	*P* value	β (95% CI)	*P* value
H1b	21.6	− 0.48 (− 2.53, 1.56)	0.64	0.10 (− 0.52, 0.72)	0.76
H1c	15.1	− 1.10 (− 3.42, 1.22)	0.36	0.00 (− 0.71, 0.71)	0.99
H1d	10.8	1.18 (− 1.51, 3.88)	0.39	0.12 (− 0.70, 0.94)	0.78
H1e	6.7	0.05 (− 3.27, 3.37)	0.98	0.38 (− 0.63, 1.38)	0.46
H1h	4.9	− 1.00 (− 4.76, 2.76)	0.60	0.08 (− 1.06, 1.23)	0.89
H1i	6.3	− 1.62 (− 5.20, 1.96)	0.38	− 1.04 (− 2.12, 0.04)	0.061
H1k^1^	1.0	2.49 (− 7.51, 12.49)	0.63	− 2.60 (− 5.61, 0.41)	0.093
H1l	3.8	1.80 (− 2.49, 6.09)	0.41	0.31 (− 1.00, 1.62)	0.64
H1m	1.7	− 4.68 (− 12.23, 2.87)	0.23	− 2.01 (− 4.30, 0.28)	0.087
H1o	4.3	− 2.72 (− 7.19, 1.75)	0.24	− 0.48 (− 1.85, 0.88)	0.49
H1p	1.1	0.94 (− 7.98, 9.85)	0.84	3.42 (0.76, 6.08)	0.013
H1q	1.2	0.08 (− 7.97, 8.12)	0.99	− 0.06 (− 2.50, 2.38)	0.96
H1r	2.1	− 0.27 (− 6.67, 6.13)	0.93	0.87 (− 1.06, 2.81)	0.38
H1s^2^	1.3	1.11 (− 6.92, 9.13)	0.79	0.29 (− 2.15, 2.73)	0.82
H1u	4.4	2.56 (− 1.28, 6.41)	0.19	0.01 (− 1.17, 1.19)	0.98
H1v	1.3	0.42 (− 7.03, 7.87)	0.91	1.79 (− 0.46, 4.03)	0.12
H1x	1.9	0.66 (− 5.57, 6.90)	0.84	− 0.20 (− 2.09, 1.69)	0.84
H2	7.7	1.23 (− 1.58, 4.04)	0.39	− 0.02 (− 0.87, 0.84)	0.97

β values, 95% CIs, and *P* values result from score tests of association that were adjusted for sex (age of CBD onset analysis) or sex and age of CBD onset (disease duration analysis). β values correspond to each additional copy of the given haplotype. *P* values < 0.0028 are considered as statistically significant after applying a Bonferroni correction for multiple testing

*CBD* corticobasal degeneration, *β* regression coefficient, *CI* confidence interval

^1^The specific haplotype for H1k was not previously shown in Table 2 as this haplotype occurred at a frequency < 1% in the overall case–control series; this haplotype is A-A-A-C-A-G for the rs1467967-rs242557-rs3785883-rs2471738-rs8070723-rs7521 haplotype

^2^The specific haplotype for H1s was not previously shown in Table 2 as this haplotype occurred at a frequency < 1% in the overall case–control series; this haplotype is G-G-G-C-A-G for the rs1467967-rs242557-rs3785883-rs2471738-rs8070723-rs7521 haplotype

## Discussion

Although there is still much to be understood regarding genetic risk factors for CBD, findings to date have demonstrated a strong association involving the *MAPT* H1 haplotype [11]. Examination of *MAPT* H1 subhaplotypes provides the opportunity to better understand any notable association involving the H1 haplotype, and indeed several studies have observed associations between *MAPT* H1 subhaplotypes and various neurodegenerative diseases [1, 9, 13, 14, 17, 24]. Herein, in addition to further confirming the strong association between the H2 haplotype and reduced risk of CBD, we have identified a novel association between the H1d subhaplotype and risk of CBD. The H1c subhaplotype also displayed some degree of association with CBD, though this was not significant after multiple testing correction (possibly due to lack of power given the relatively small sample size). No strong associations of *MAPT* H1 subhaplotypes with severity of tau pathology, age of CBD onset, or disease duration were noted. Though replication will be crucial, these findings suggest that several specific *MAPT* H1 subhaplotypes (H1d and H1c) are primarily responsible for the strong association between *MAPT* H1 and risk of CBD, but that H1 subhaplotypes are unlikely to play a major role in driving severity of tau pathology or clinical features.

Only one previous study has assessed associations between *MAPT* subhaplotypes and risk of CBD, where Pittman et al. [17] examined a series of 44 CBD cases and 131 controls. Their results are generally in line with the findings of our current study; H2 was associated with a significantly decreased risk of CBD (8.2% vs. 22.0%, *P* = 0.020), and although not statistically significant in this small series with very low power to detect associations, both H1c (17.7% vs. 7.8%, *P* = 0.066) and H1d (7.5% vs. 4.0%, *P* = 0.49) were observed more frequently in CBD cases than controls. The authors also observed a nominally significant association between H1n and a decreased risk of CBD (0.0% vs. 4.3%, *P* = 0.018). The H1n haplotype was observed in only 0.6% of all subjects in our case–control series and therefore was not formally assessed in disease-association analysis where only haplotypes that were observed at a frequency of at least 1% in the overall series were assessed. Further examination of the H1n subhaplotype in our series reveals a frequency of 0.0% in CBD cases and 0.6% in controls, which does not replicate the previously observed nominally significant finding. Overall, the results of the study by Pittman et al. support those of our current study, which implicates the H1c and H1d *MAPT* H1 subhaplotypes in susceptibility to CBD.

The results of our disease-association haplotype analysis are somewhat in line with those of a previous study by our group involving PSP [9], which is not surprising given the similar clinical and neuropathological features of CBD and PSP. More specifically, with a sample size of 802 PSP cases and 1312 controls (the same control group examined in the current study), the *MAPT* H2 haplotype was more strongly associated with PSP (OR = 0.16, *P* = 7 × 10^−49^) than CBD (OR = 0.26, *P* = 2 × 10^−12^), H1d was significantly associated with an increased risk of PSP (OR = 1.86, *P* = 2 × 10^−6^) with a similar effect size compared to CBD (OR = 1.76, *P* = 0.002), while H1c was also more strongly associated with PSP risk (OR = 2.15, *P* = 2 × 10^−14^) than it was with CBD (OR = 1.49, *P* = 0.009). Additionally, H1o was associated with a significantly higher risk of PSP (OR = 2.60, *P* = 2 × 10^−5^), which is not overly divergent from the non-significant OR of 1.81 (*P* = 0.080) that we noted for CBD; the absence of a statistically significant association between *MAPT* H1o and risk of CBD in our study may be due to the less precise haplotype frequencies and lower power to detect associations that we had in comparison to our previous PSP study where the number of cases was greater than threefold higher. Though weaker evidence, the H1i subhaplotype was nominally associated with increased risk in both CBD (OR = 1.65, *P* = 0.047) and PSP (OR = 1.56, *P* = 0.02). Conversely, the H1g subhaplotype was a significant risk factor for PSP (OR = 3.64, *P* = 2 × 10^−6^) but displayed very little evidence of an association with CBD (OR = 1.45, *P* = 0.47), while H1b was nominally associated with CBD (OR = 1.31, *P* = 0.049) but not PSP (OR = 1.06, *P* = 0.57). Larger series will be needed to further confirm the suggestive association between H1c and CBD that we observed, as well as to examine whether the H1o subhaplotype may be a genetic risk factor for CBD as it is for PSP. Overall, our findings suggest that although similarities in *MAPT* haplotype risk factor profile exist between these two primary 4R-tauopathies, with H2, H1d, and H1c displaying associations with both diseases, the strength of association may be stronger for PSP as evidenced by generally weaker association ORs in CBD for the three aforementioned shared susceptibility haplotypes, and a lack of strong association with CBD for several PSP risk haplotypes (H1g and H1o).

As previously mentioned, in the previous CBD GWAS, there was suggestive evidence of an association between the rs242557 variant that partially tags the *MAPT* H1c haplotype (OR = 1.57, *P* = 7.9 × 10^−6^) [11]. Interestingly, though our haplotype-specific analysis mostly supports this finding as the rs242557 risk allele (A) is observed for the H1c, H1d, H1i, and H1o subhaplotypes (all of which were associated with an increased risk of CBD with a *P* value < 0.10), findings differ for the H1b haplotype. H1b is the most common H1 subhaplotype and is associated with the protective (G) allele of rs242557; the nominally significant evidence of an association between H1b and an increased risk of CBD that we observed demonstrates that rs242557 is not consistently associated with CBD risk, and underscores the importance of assessing specific H1 subhaplotypes when examining the role of *MAPT* in susceptibility to CBD.

Interestingly, despite the somewhat similar findings regarding the role of *MAPT* haplotypes in susceptibility to CBD and PSP, findings differ regarding associations with severity of tau pathology. This is most evident when considering the three shared protective (H2) and risk (H1c and H1d) haplotypes between these two tauopathies; all three of these haplotypes were correlated with severity of tau pathology in PSP cases in the aforementioned previous study by our group, where small but nominally significant associations were observed [9]. Conversely, the *MAPT* H2, H1c, and H1d haplotypes were not significantly associated with severity of tau pathology in our current study of CBD cases. Of course, one explanation for this could be the aforementioned much lower power we had in this study to detect associations with severity of tau pathology in CBD. Correspondingly, it may be noteworthy that H1c was associated with severity of CB tau pathology with a p-value of 0.073 and an effect size of 0.08 that is larger than any of those observed in PSP where nominal significance was reached for this haplotype (effect sizes of 0.05 were observed for severity of both CB and tufted astrocytes tau pathology). Along these lines, the smaller degree of variability in overall tau pathology scores (NT in particular) that we observed for CBD in comparison to PSP [9] would also have limited power to detect associations with these measures. Nonetheless, it is clear that regardless of the presence or absence of any statistically significant findings, no *MAPT* haplotypes are strongly associated with neuropathologic severity of disease in CBD or PSP. Additionally, our current findings provide further support for the hypothesis that H1 subhaplotypes are not major disease modifiers in CBD, as no significant associations with age of CBD onset or disease duration were observed.

Several limitations of our study are important to bear in mind. First, as previously referred to, the sample size is relatively small, and therefore the possibility of a type II error (i.e. false-negative finding) is important to consider, particularly for rarer haplotypes and after multiple testing adjustment. Second, without available genome-wide control markers that would have allowed us to adjust regression models for top principal components, we cannot exclude the possibility that population stratification could have influenced our findings. Finally, our study did not include a replication series, and as a result validation of our findings will be important. However, it is worth noting that the haplotype frequencies observed in our series of 1312 controls are similar to those seen in a large independent control cohort of 8144 European-American subjects from the Alzheimer’s Disease Genetics Consortium (ADGC) in a previous study by Allen et al [1]. Specifically, when comparing our 1312 Mayo Clinic controls to those 8144 ADGC controls, similar haplotype frequencies were observed for H2 (22.7% vs. 23.6%), H1d (7.1% vs. 7.9%), and to a slightly lesser extent H1c (11.3% vs. 13.2%).

## Conclusions

Due to the rare nature of CBD, meta-analytic studies will be needed to gain a solid understanding of genetic risk factors. In addition to variation at *MAPT*, the initial CBD GWAS highlighted *lnc*-*KIF13B*-*1* as well as possibly *MOBP* and *SOS1* as susceptibility loci. Additionally, findings of a recent study suggest that mitochondrial genomic background may be associated with risk of CBD [23]. The results of our current study add to the understanding of the genetic etiology of this rare tauopathy by shedding further light on the strongest genetic risk factor for CBD that has been observed thus far—the *MAPT* H1 haplotype. Specifically, our results suggest that this association is driven primarily by the H1d and H1c haplotypes, but that *MAPT* haplotypes likely do not play a major role in clinical or neuropathologic presentation of CBD. Future replication studies that directly account for population stratification will be important to confirm our initial findings.

## Supplementary Information

**Additional file 1** Supplemental materials.

## Data Availability

The datasets generated and/or analyzed during the current study are available from the corresponding author on reasonable request.

## Abbreviations

CBD: Corticobasal degeneration
PSP: Progressive supranuclear palsy
AD: Alzheimer’s disease
FTD: Frontotemporal dementia
NFT: Neurofibrillary tangles
NT: Neuropil threads
AP: Astrocytic plaques
CB: Oligodendroglial coiled bodies
4R: Four-repeat tau isoform
MAPT: Microtubule-associated protein tau
GWAS: Genome-wide association study
MCSA: Mayo Clinic Study of Aging
ADRC: Alzheimer’s disease Research Center
OR: Odds ratio
CI: Confidence interval
