# Supplementary material for: MAPT subhaplotypes in corticobasal degeneration: assessing associations with disease risk, severity of tau pathology, and clinical features
Source: Acta Neuropathol Commun. 2020 Dec 7;8:218. doi: 10.1186/s40478-020-01097-z (PMC7720600; doi:10.1186/s40478-020-01097-z)
Supplement: Supplementary file 1 — Additional file 1 Supplemental materials. [file 40478_2020_1097_MOESM1_ESM.docx]

**Supplementary Table 1: Genotype counts and frequencies**

| Variant | Minor allele count and frequency | Major allele count and frequency | Genotype 1 count and frequency | Genotype 2 count and frequency | Genotype 3 count and frequency |
| --- | --- | --- | --- | --- | --- |
| rs1467967 |  |  |  |  |  |
| CBD patients | G: 169 (36.7%) | A: 21 (63.3%) | AA: 91 (39.6%) | AG: 109 (47.4%) | GG: 30 (13.0%) |
| Controls | G: 849 (32.4%) | A: 1775 (67.6%) | AA: 603 (46.0%) | AG: 569 (43.4%) | GG: 140 (10.7%) |
| rs242557 |  |  |  |  |  |
| CBD patients | A: 226 (49.1%) | G: 234 (50.9%) | GG: 61 (26.5%) | GA: 112 (48.7%) | AA: 57 (24.8%) |
| Controls | A: 965 (36.8%) | G: 1659 (63.2%) | GG: 548 (41.8%) | GA: 563 (42.9%) | AA: 201 (15.3%) |
| rs3785883 |  |  |  |  |  |
| CBD patients | A: 89 (19.3%) | G: 371 (80.7%) | GG: 150 (65.2%) | GA: 71 (30.9%) | AA: 9 (3.9%) |
| Controls | A: 472 (18.0%) | G: 2152 (82.0%) | GG: 879 (67.0%) | GA: 394 (30.0%) | AA: 39 (3.0%) |
| rs2471738 |  |  |  |  |  |
| CBD patients | T: 118 (25.7%) | C: 342 (74.3%) | CC: 127 (55.2%) | CT: 88 (38.3%) | TT: 15 (6.5%) |
| Controls | T: 542 (20.7%) | C: 2082 (79.3%) | CC: 842 (64.2%) | CT: 398 (30.3%) | TT: 72 (5.5%) |
| rs8070723 |  |  |  |  |  |
| CBD patients | G: 34 (7.4%) | A: 426 (92.6%) | AA: 199 (86.5%) | AG: 28 (12.2%) | GG: 3 (1.3%) |
| Controls | G: 603 (23.0%) | A: 2021 (77.0%) | AA: 784 (59.8%) | AG: 453 (34.5%) | GG: 75 (5.7%) |
| rs7521 |  |  |  |  |  |
| CBD patients | A: 262 (57.0%) | G: 198 (43.0%) | AA: 80 (34.8%) | AG: 102 (44.3%) | GG: 48 (20.9%) |
| Controls | A: 1223 (46.6%) | G: 1401 (53.4%) | GG: 385 (29.3%) | GA: 631 (48.1%) | AA: 296 (22.6%) |

**Supplementary Table 2: Neuroanatomical regions that are vulnerable to CBD that were assessed for severity of tau pathology**

| Neuroanatomical region |
| --- |
| Superior frontal gyrus |
| Motor cortex |
| Temporal cortex |
| Caudate putamen |
| Globus pallidus |
| Basal nucleus |
| Hypothalamus |
| Subthalamic nucleus |
| Thalamic fasciculus |
| Ventral thalamus |
| Midbrain tectum |
| Oculomotor complex |
| Red nucleus |
| Substantia nigra |
| Locus ceruleus |
| Pontine tegmentum |
| Pontine base |
| Medullary tegmentum |
| Inferior olive |
| Cerebellar white matter |
| Dentate nucleus |
